# Effect of ablated hippocampal neurogenesis on the formation and extinction of contextual fear memory

**DOI:** 10.1186/1756-6606-2-1

**Published:** 2009-01-13

**Authors:** Hyoung-Gon Ko, Deok-Jin Jang, Junehee Son, Chuljung Kwak, Jun-Hyeok Choi, Young-Hoon Ji, Yun-Sil Lee, Hyeon Son, Bong-Kiun Kaang

**Affiliations:** 1 National Creative Research Initiative Center for Memory, Department of Biological Sciences, College of Natural Sciences, Seoul National University, San 56-1 Silim-dong, Gwanak-gu, Seoul, 151-747, Korea; 2 Laboratory of Radiation Effect, Department of Radiation Oncology, Korea Institute of Radiological and Medical Sciences, 139-706, Korea; 3 Division of Radiation Cancer Research, Korea Institute of Radiological and Medical Sciences, 139-706, Korea; 4 Departments of Biochemistry and Molecular Biology, Hanyang University College of Medicine, 17 Haengdang-dong, Sungdong-gu, Seoul 133-791, Korea

## Abstract

Newborn neurons in the subgranular zone (SGZ) of the hippocampus incorporate into the dentate gyrus and mature. Numerous studies have focused on hippocampal neurogenesis because of its importance in learning and memory. However, it is largely unknown whether hippocampal neurogenesis is involved in memory extinction *per se*. Here, we sought to examine the possibility that hippocampal neurogenesis may play a critical role in the formation and extinction of hippocampus-dependent contextual fear memory. By methylazoxymethanol acetate (MAM) or gamma-ray irradiation, hippocampal neurogenesis was impaired in adult mice. Under our experimental conditions, only a severe impairment of hippocampal neurogenesis inhibited the formation of contextual fear memory. However, the extinction of contextual fear memory was not affected. These results suggest that although adult newborn neurons contribute to contextual fear memory, they may not be involved in the extinction or erasure of hippocampus-dependent contextual fear memory.

## Background

Newborn neurons continuously incorporate into neuronal circuits during development. In the adult brain, neurogenesis occurs in the subgranular zone (SGZ) of the hippocampus and the subventricular zone (SVZ) of the lateral ventricle [1]. Newly generated neurons in the SVZ migrate to the olfactory bulb and play key roles in olfactory memory [2]. In the SGZ region, newly generated cells are differentiated into both neurons and glial cells, and the newborn neurons incorporate to the granule cell layer of the dentate gyrus [2]. A growing body of evidence supports the contribution of hippocampal newborn neurons to hippocampus-dependent memories [3-5]. It was reported that neurogenesis in the adult hippocampus is required for trace eyeblink conditioning [4]. Moreover, ablated neurogenesis impaired contextual fear conditioning, but did not impair cued fear conditioning, which is independent of the hippocampus [3,5]. These phenomena are well correlated with electrophysiological data showing reduced long-term potentiation (LTP) in the dentate gyrus owing to impaired hippocampal neurogenesis [3]. Therefore, many studies support the idea that hippocampal neurogenesis is involved in the acquisition or expression of hippocampus-dependent memories (Table 1) [3-8]. In contrast, some reports indicate that ablation of hippocampal neurogenesis has no effect on learning, even in hippocampus-dependent memory tasks such as the contextual fear memory task and the Morris water maze task (Table 1) [3-6,8,9].

**Table 1:** Effects of ablated neurogenesis on learning and memory.

**Ablation method**	**Extent of ablation**	**Behavior task tested**	**Species (Strain)**	**Effect**	**Ref**
MAM	~84%	Trace eye blink conditioning	Rat (Sprague-Dawley)	-	[4]
	~84%	Delayed eye blink conditioning	Rat (Sprague-Dawley)	·	[4]
	~75%	Morris water maze	Rat (Sprague-Dawley)	·	[6]
	~85%	Trace fear conditioning	Rat (Sprague-Dawley)	-	[6]
	~85%	Contextual fear memory	Rat (Sprague-Dawley)	·	[6]
Irradiation	~85% *	Contextual fear memory	Mouse (129SvEv)	-	[3]
	~85% *	Cued fear memory, Morris water maze, Y maze	Mouse (129SvEv)	·	[3]
	~75%	Radial arm maze (hippocampus dependent)	Mouse (129SvEv)	+	[7]
	~90%	Morris water maze	Rat (Long Evans)	·	[8]
	~90%	Morris water maze (only long-term memory)	Rat (Long Evans)	-	[8]
Transgenic	~85% *	Contextual fear memory	Mouse (C57BL6-BALBc mixed)	-	[3]
	~85% *	Cued fear memory	Mouse (C57BL6-BALBc mixed)	·	[3]
	~75%	Radial arm maze (hippocampus dependent)	Mouse (C57BL6-BALBc mixed)	+	[7]
	N. S.	Barnes maze, Contextual fear memory	Mouse (C57BL/6J)	-	[5]
	N. S.	Cued fear memory	Mouse (C57BL/6J)	·	[5]
	~66%	Contextual fear memory, Cued fear memory	Mouse (backcrossing to C57BL/6J)	·	[9]

Many studies have reported a relationship between hippocampal neurogenesis and memory. This table summarizes debates about the role of neurogenesis in learning and memory. N.S., not stated in paper; *, inferred from previous paper; +/•/-, ablation of neurogenesis enhances/has no effect on/impairs the described task.

Recently, several reports have shown that the hippocampus is involved in the extinction of hippocampus-dependent memories [10,11]. Usually, extinction can be explained by two possible mechanisms. First, memory extinction is an active learning process [12]. Therefore, the expression of old memories can be inhibited by the increase of synaptic strength of inhibitory circuits, which can be regulated by other brain regions, including the prefrontal cortex. In case of extinction of cued fear memory, for example, infralimbic cortex activates inhibitory interneurons in the amygdala [13]. As a result of fear extinction, this inhibitory action suppresses the fear response mediated by the amygdala. Moreover, this theory is supported by several recovery effects such as renewal, spontaneous recovery, and reinstatement after extinction [12]. Second, old memories can be extinguished by erasing preexisting circuits. A growing body of evidence suggests that synaptic alteration in preexisting circuits plays a key role in inducing memory extinction. For example, it was recently reported that synaptic depotentiation is directly involved in cued fear extinction in the amygdala [14]. In addition, ubiquitin- and proteasome-dependent protein degradation is required for contextual fear extinction in hippocampus [15]. However, it has not been examined whether the incorporation of newborn neurons into the hippocampus is involved in memory extinction. Here, we directly interfered with hippocampal neurogenesis through MAM or gamma-ray irradiation to examine the effects of the ablation of hippocampal neurogenesis on contextual fear memory formation and extinction.

## Results

In order to examine the role of neurogenesis during memory formation and extinction, adult male mice were subcutaneously injected with several doses of MAM (1, 2.5 and 5 mg/kg) for 2 weeks. Since MAM can induce cachexsia, we monitored the general health and weight of the injected mice [16]. We did not detect any weight loss or fur deterioration in the MAM-treated group. Instead, retardation of weight gain was detected in the mice that were injected with 2.5 mg/kg MAM (vehicle vs. 2.5 mg/kg, p < 0.05, data not shown). However, the 2.5 mg/kg MAM-treated mice showed similar levels of moving distance in the open field test (data not shown).

**Figure 1:**
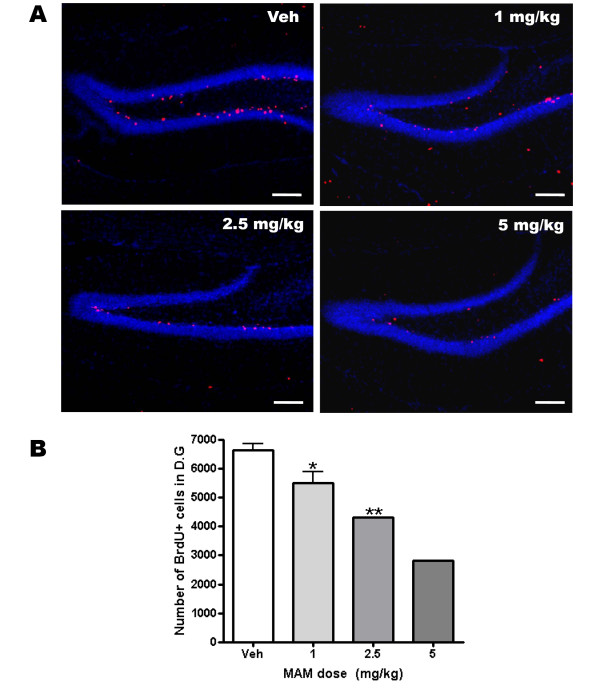
**MAM treatments reduced hippocampal neurogenesis but did not affect gross morphology**. (A) Representative images show the dentate gyrus of mice that were injected with MAM for 2 weeks. Doses of MAM are indicated in each image. Cells were labeled with BrdU (red) and DAPI (blue). Upper left: vehicle (Veh). Upper right: 1 mg/kg. Lower left: 2.5 mg/kg. Lower right: 5 mg/kg. Scale bar, 100 μm. (B) Total number of BrdU+ cells in dentate gyrus. Three mice were used for analysis. Note that two of the three mice died during 5 mg/kg MAM injection. *, p < 0.05; **, p < 0.01 compared with vehicle. Dentate gyrus (D.G).

After the open field test, the mice were sacrificed for BrdU immunohistochemistry. MAM-treated dentate gyrus showed normal morphology compared with the vehicle-treated dentate gyrus (Fig. 1A). However, in MAM-treated mice, the number of BrdU+ cells was decreased in a dose-dependent manner. MAM treatments reduced the number of BrdU+ cells in the dentate gyrus by 17%, 35% and 58% in 1 mg/kg, 2.5 mg/kg and 5 mg/kg, respectively (Fig. 1B; n = 3 except for 5 mg/kg, n = 1; *, p < 0.05; **, p < 0.01 compared with vehicle). Notably, two out of three mice in the 5 mg/kg group died during MAM treatment. Therefore, we selected 3 mg/kg as MAM dose for the following behavioral experiments.

**Figure 2:**
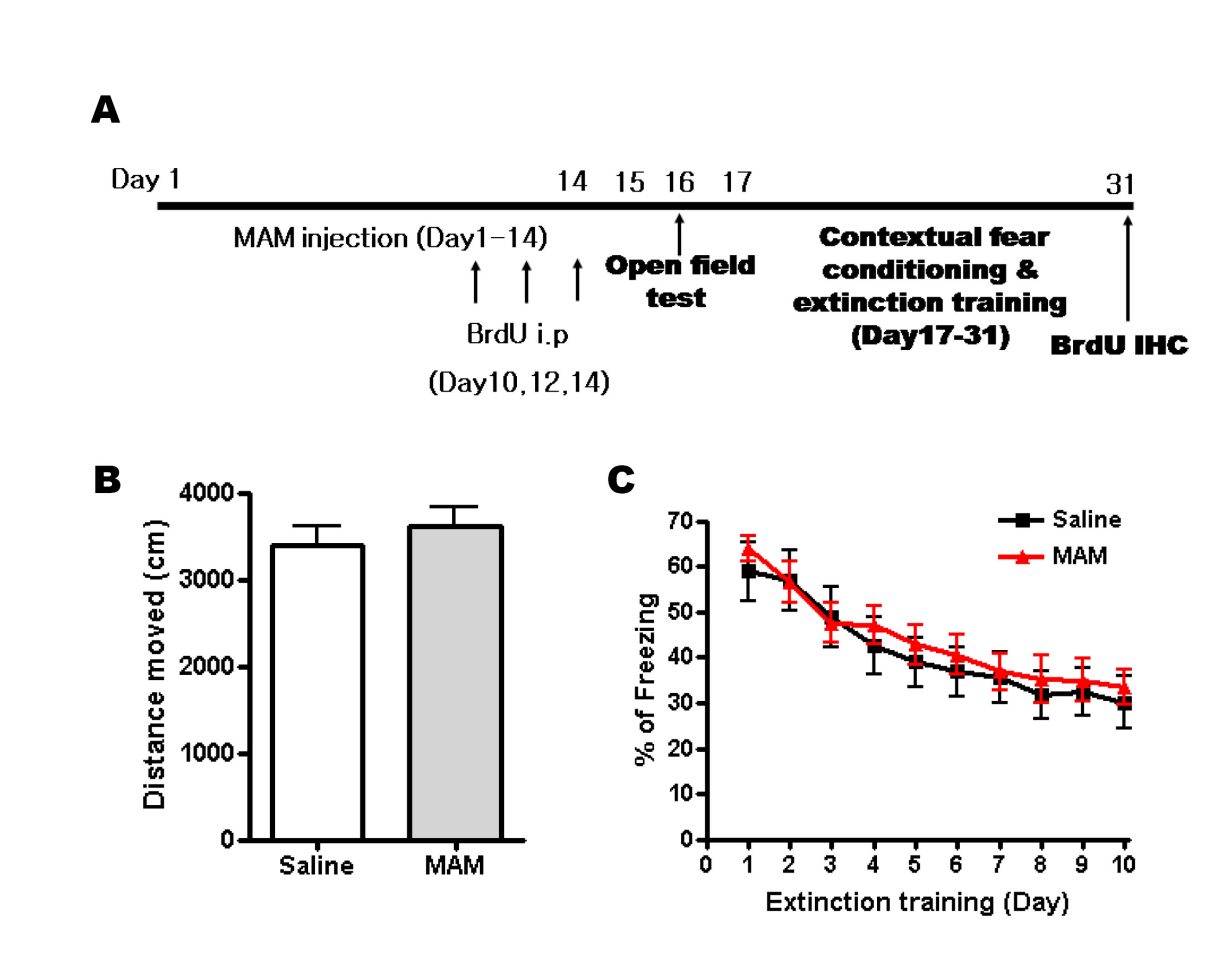
**Reduced hippocampal neurogenesis did not impair locomotion or the extinction of contextual fear memory**. (A) A schematic diagram showing the experimental procedure using MAM. (B) Total distance moved of the saline- and MAM-treated mice. (C) Percentage of freezing across 10 days of extinction training in mice, treated with either saline or MAM.

As shown in Fig. 2A, the open field test, contextual fear conditioning and extinction training were performed serially. Finally, the proliferation of newborn neurons was examined with immunohistochemistry. In the open field test, there was no difference between groups in the distance moved (Fig. 2B; saline = 3,390 cm ± 218 cm, MAM = 3,616 cm ± 225 cm, n = 10 and 13, respectively, p > 0.05). This result shows that 3 mg/kg of MAM did not affect general locomotion of the mice. When the MAM-treated mice were trained with contextual fear conditioning, they showed a similar percentage of freezing level on the following day (Fig. 2C; saline = 58.9% ± 6.5%, MAM: 64.0% ± 2.7%, n = 10 and 13, respectively, p > 0.05). Furthermore, ANOVA analysis revealed that MAM had no effect on extinction (p > 0.05). These results suggest that the depletion of newborn neurons did not affect either the formation or the extinction of contextual fear memory. However, when we performed BrdU immunohistochemistry to confirm the depletion of newborn neurons by MAM treatment, we did not find a significant reduction in the total number of BrdU+ cells in the MAM-treated dentate gyrus (data not shown). Since the mice were decapitated 17 days after the BrdU injection, it is possible that the survival of newborn neurons in MAM-treated mice may be enhanced by maintaining the proper number of newborn neurons.

**Figure 3:**
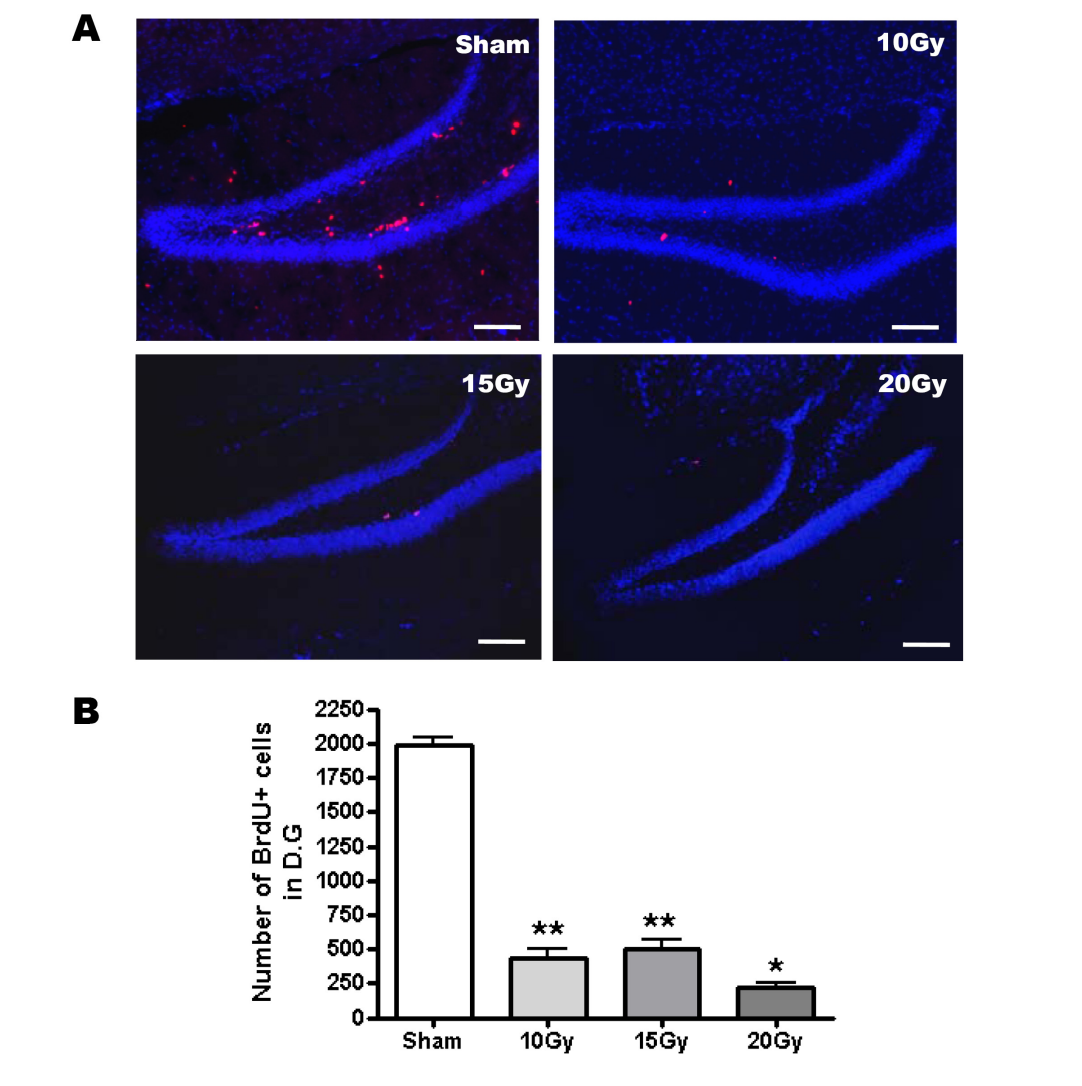
**Gamma-ray irradiation ablated hippocampal neurogenesis**. (A) Each image shows the dentate gyrus of sham (upper) and gamma-ray irradiated (lower) mice. Proliferating cells were stained with BrdU (red). Nuclei were counterstained with DAPI (blue). Scale bar, 100 μm. (B) Total number of BrdU+ cells in dentate gyrus of sham, 10 Gy-, 15 Gy- and 20 Gy-irradiated mice. **, p < 0.01 compared with sham; *, p < 0.05 compared with 10 Gy.

**Figure 4:**
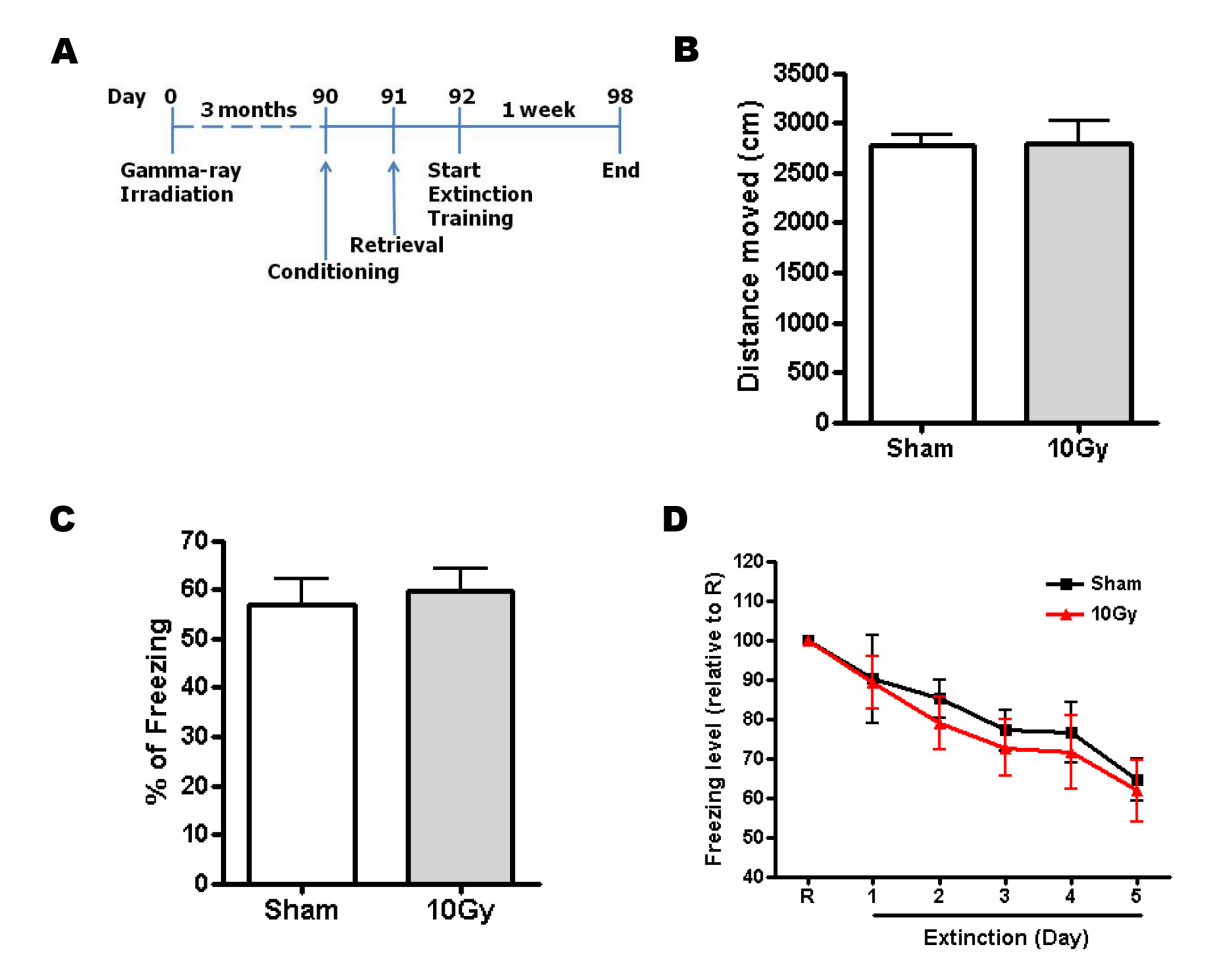
**Low gamma-ray irradiation did not block the formation and extinction of contextual fear conditioning with a 24-hour interval between retrieval and extinction training**. (A) Experimental procedures. (B) Total distance moved of sham and 10 Gy-irradiated mice. (C) Percent freezing in sham and 10 Gy-irradiated group on the retrieval day. (D) Percent freezing was presented with value relative to it on the retrieval day.

To clearly address the effects of neurogenesis deficiency on the formation and extinction of contextual fear memory, we used gamma-ray irradiation, which is known to ablate newborn neurons more completely than MAM treatment [17]. The latter is known to only reduce newborn neurons by up to 35%. Only the hippocampal region was exposed to gamma rays, while other brain regions were shielded from irradiation [17,18]. We performed behavioral experiments 3 months after the irradiation in order to avoid side effects such as inflammation by activation of the microglia [19]. After behavioral experiments, brain slices were analyzed with immunohistochemistry. One gamma-ray application depleted the number of BrdU+ cells in the dentate gyrus more efficiently than MAM treatments. Irradiated dentate gyrus showed a reduction of newborn neurons by 78%, 75% and 89% in 10 Gy, 15 Gy and 20 Gy, respectively (Fig. 3B; sham = 1,988 ± 58; 10 Gy = 433 ± 70; 15 Gy = 499 ± 73; 20 Gy = 216 ± 35; n = 8–16; **, p < 0.001 compared with sham; *, p < 0.05 compared with 15 Gy). However, we found no abnormal morphological features in the dentate gyrus from irradiated group (Fig. 3A). Moreover, the total distance moved showed no difference between sham and 10 Gy (Fig. 4B; sham = 2,770 cm ± 112 cm; 10 Gy = 2,797 cm ± 226 cm, n = 9, p > 0.05).

To examine the effect of ablated neurogenesis on the formation and extinction of contextual fear memory, we used a procedure similar to that of the MAM experiments. There was a 24-hour interval between retrieval and extinction training (Fig. 4A). In consistent with the MAM results, 10 Gy irradiation had no effect on the formation and extinction of fear conditioning compared with the sham control (Fig. 4C, D; n = 3–6, p > 0.05).

**Figure 5:**
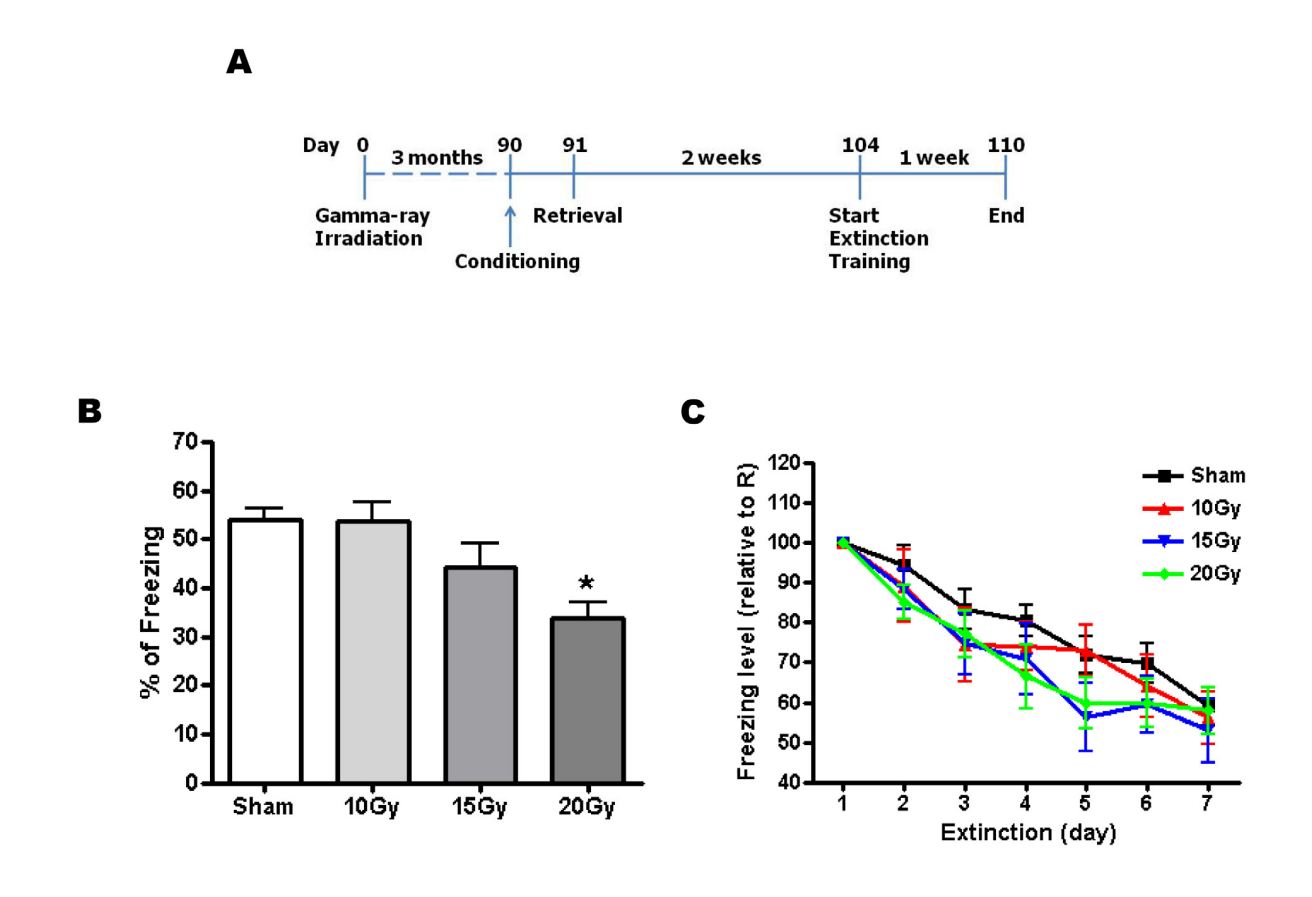
**High gamma-ray irradiation blocked the formation but not the extinction of contextual fear memory with a 2-week interval between retrieval and extinction training**. (A) Experimental procedures. (B) Percent freezing in gamma-ray-irradiated groups on the retrieval day. 20 Gy-irradiated group showed less freezing than sham or 10 Gy (*, p < 0.05). (C) Percent freezing was presented with value relative to it on the retrieval day. R; retrieval.

**Figure 6:**
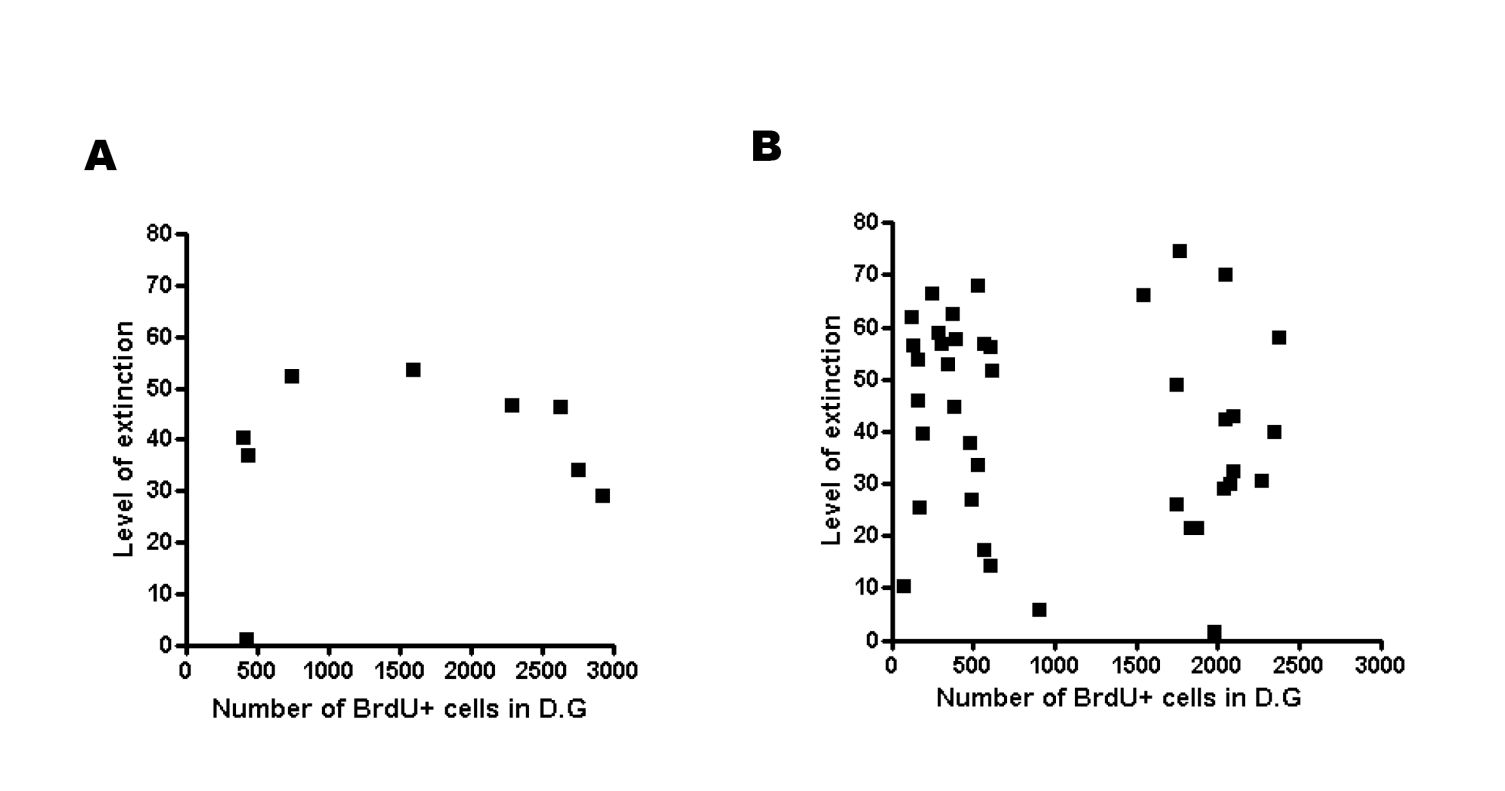
**Level of extinction is not correlated with total number of BrdU+ cells**. (A) and (B) Level of extinction is not correlated with total number of BrdU+ cells. Data in Figs. 4D and 5C were reanalyzed to examine the correlation between the level of extinction and the level of hippocampal neurogenesis. Level of extinction was calculated by (R – the first freezing)/R × 100% in case of (A), (the first freezing – the seventh freezing)/the first freezing × 100% in case of (B). Dentate gyrus (D.G).

Next, we speculated that if hippocampal neurogenesis is required for extinction, the incorporation of newborn neurons in the dentate gyrus circuit may be important because it could interfere with the flow of information that encodes contextual fear memory [20]. Therefore, in the next experiment, we introduced a 2-week interval between retrieval and extinction training because some contents of the synaptic connection may be required for blocking the flow of old memory (Fig. 5A) [1]. As shown in Fig. 5B, 20 Gy-irradiated mice showed less freezing compared with sham or 10 Gy when they were reexposed to the conditioned context on the day of retrieval (Fig. 5B, sham = 54.0% ± 2.5%; 10 Gy = 53.8% ± 3.9%; 15 Gy = 44.3% ± 4.8%; 20 Gy = 33.9% ± 3.4%; n = 8–16; *, p < 0.05 compared with sham or 10 Gy). However, gamma-ray application had no effect on extinction (Fig. 5C; n = 8–16, p > 0.05). These results are consistent with Fig. 2C. Reduced neurogenesis did not impair the extinction of contextual fear memory. To demonstrate these results clearly, we further analyzed the data from gamma-ray irradiation experiments. The level of extinction was plotted against the number of BrdU+ cells in the dentate gyrus. However, we did not find any correlation between the level of extinction and the level of neurogenesis (Fig. 6A, B; A: r = 0.194, p > 0.05; B: r = -0.282, p > 0.05). These data suggest that hippocampal neurogenesis is not correlated with the extinction of contextual fear memory. Considering that MAM treatment and low gamma-ray (10 Gy and 15 Gy) irradiation did not affect freezing, these results suggested that newborn neurons should be reduced by approximately 89% in order to significantly inhibit the formation of contextual fear memory.

## Discussion and conclusion

Since newborn neurons show a high capacity of synaptic plasticity and incorporate into preexisting circuits, it is conceivable that adult neurogenesis may contribute to building up or impairing the circuitry for memory storage [21,22]. In this study, we examined the effect of hippocampal neurogenesis on the formation and extinction of a hippocampus-dependent contextual fear memory.

When hippocampal neurogenesis was ablated by MAM treatment or gamma-ray irradiation, the retrieval of contextual fear memory was affected when newborn neurons were severely reduced by approximately 89% (Fig. 1, 2C, 3 and 4D). This result is consistent with other ablation studies showing that newborn neurons were required for contextual fear conditioning. However, Zhang et al. found no effect of the depletion of neurogenesis on contextual fear conditioning (Table 1) [9]. This could be explained by the fact that hippocampal neurogenesis was only reduced by a level of 66%. In these studies, such a reduction may be insufficient to affect contextual fear conditioning. Our results did not indicate any effect of ablated newborn neurons on the same learning task except for the 20 Gy-irradiated group, in which neurogenesis was reduced by approximately 89%. Consistent with our result, it was recently shown that ablation of neurogenesis for 6 months which could significantly reduce the density of the granule cells in the dentate gyrus, impaired contextual fear conditioning [5]. Thus, it is plausible that a small fraction of newborn neurons are enough to play a functional role in the formation of contextual fear memory.

It has been reported that adult neurogenesis in the dentate gyrus may be involved in the processing of memory clearance [20,23]. In conditional presenilin-1 knockout mice, enrichment-induced neurogenesis, but not basal neurogenesis, was deficient. This impairment increased contextual fear conditioning because it blocked memory clearance in the hippocampus [23]. However, there was no direct correlation between fear extinction and adult neurogenesis. Therefore, it is still unclear whether hippocampal neurogenesis is involved in fear extinction. Theoretically, it could be predicted that if newborn neurons incorporated into the preexisting neural circuits of the dentate gyrus during fear memory processing, retrieval of fear memory would be impaired because the flow of information encoding contextual fear memory would be blocked by the incorporation of newborn neurons. Moreover, newly generated neurons in the dentate gyrus rapidly make new synapses into the CA3 region [24]. Therefore, it would be interesting to determine whether adult neurogenesis could be involved in fear extinction of preexisting memory. To test this idea, after introducing 24-hour or 2-week intervals between retrieval and fear extinction training, respectively, we compared the effects of the incorporation of newborn neurons into the preexisting circuits. However, in all irradiation groups, including the 20 Gy-irradiated group in which fear conditioning was affected to some degree, no effect was observed on the fear extinction (Figs. 2, 4, and 5). Thus, adult neurogenesis may not be involved in fear extinction, though more complicated extinction processes may require neurogenesis. Fear extinction is a quite complicated processing in which an active learning and erasing of existing memory are involved. Therefore, further studies may be required to clearly elucidate the correlation between memory extinction and hippocampal neurogenesis.

## Methods

### Subjects

Male C57BL/6NCrljBgi mice aged between 6 and 8 weeks were purchased from Orient Bio (Korea). Animals were housed in standard laboratory cages on a 12-hour light-dark cycle and provided with access to food and water *ad libitum*. Mice were used for all experiments 1–2 weeks after being housed in the laboratory cage.

### Drug injection

MAM (MRI, F0040) was diluted with saline and delivered daily to mice subcutaneously for 2 weeks. For testing the proper dose of MAM, three mice were used for 1, 2.5, 5 mg/kg each (Fig. 1). In behavioral experiments, 3 mg/kg was selected as the dose of MAM injection. BrdU (50 mg/kg; Sigma) was injected three times intraperitoneally on days 10, 12, and 14 during the 2 weeks of MAM treatment. For gamma-ray irradiation experiments, mice were daily injected with BrdU for 3 days after finishing behavioral experiments.

### Irradiation

Gamma-ray irradiation was performed essentially as described previously [13,14]. Mice were anesthetized with a mixture of ketamine and xylazine in saline solution. The hippocampal region of the mice was exposed to radiation emitted from a Co-60 source (AECL, Theratron-780, Canada) for 8.7 minutes, 17.6 minutes, and 23.5 minutes. This timing allowed for the delivery of a total of 10 Gy, 15 Gy, and 20 Gy dose, respectively. The radiation field size was 10 × 10 cm^2 ^and the collimator was set to 0.5 × 3 cm^2 ^around the hippocampal region. Other brain regions and body parts were shielded from radiation.

### Behavioral procedures

Two days after the MAM injection, mice performed open field tests. Mice were gently placed in a corner of the arena (40 × 40 cm white field surrounded by a 40 cm high white wall, dim light) for 10 minutes. Total distance moved was automatically recorded by the Ethovision system (Noldus Information Technology). In gamma-ray irradiation experiments, the open field test was performed one day after the end of the extinction training.

For contextual fear conditioning, mice were handled daily for 2 minutes for 4 days. On the conditioning day, mice were placed in the conditioning chamber with a stainless grid floor, and an electrical footshock (0.6 mA: MAM experiments or 0.8 mA: gamma-ray experiments, one time, 2 s) was delivered 2 minutes and 28 seconds after entry to the chamber. Thirty seconds after the shock, mice were removed from the conditioning chamber. As seen in Fig. 4D, mice were reexposed in the conditioned chamber for 5 minutes to measure the contextual fear memory through the freezing behavior. In all the other experiments, mice were placed in the conditioning chamber for 10 minutes at retrieval. For extinction training, mice were placed in the conditioning chamber for 10 minutes. The behavior of the mice was automatically recorded by a digital camera and the Freezeframe software (Coulbourn Instruments). Freezing behavior was analyzed by the Freezeview software. Setting values were 0.5 second for bouts and 11 for threshold.

### BrdU immunohistochemistry

Mice were perfused with phosphate-buffered saline (PBS) and 4% paraformaldehyde in PBS. After the 12-hour postfixation period, the brains were dehydrated in 30% sucrose in PBS overnight. Thirty μm sections were made by cryostat (Leica) in -20°C and placed in 50% glycerol in PBS. Until immunohistochemistry could be performed, slices were placed in -20°C. Slices were washed three times with PBS for 5 minutes at room temperature. For the detection of BrdU, the slices were treated with 1 N HCl at 37°C for 30 minutes and neutralized by a wash with 0.1 M sodium borate buffer for 15 minutes. Subsequently, slices were incubated with blocking solution (0.2% Triton X-100, 2% goat serum and 1% BSA in PBS) for 1 hour. After blocking, the slices in blocking solution with a BrdU antibody (1:300, rat; Abcam) were incubated overnight. After washing with PBS three times for 5 minutres, the slices were incubated with the Cy3-conjugated anti-rat IgG antibody (1:300, goat; Jackson ImmunoResearch) for 2 hours. After washing with PBS three times for 5 minutes, slices were mounted with VECTASHIELD containing DAPI (Vector Laboratories).

### Stereology

The number of BrdU+ cells in the dentate gyrus of the hippocampus was estimated with a modified optical fractionator method. Every sixth section was counted along the rostrocaudal axis of the hippocampus under a ×40 objective on a fluorescence microscope (IX51, Olympus). Images were acquired through a ×10 objective using the Wasabi software and a digital camera (Hamamatsu Photonics).

### Statistical analysis

Reduction of newborn neurons and freezing in retrieval were analyzed using one-way ANOVA followed by Tukey's multiple comparison post hoc test. Repeated measures two-way ANOVAs were used to analyze the effect of irradiation dose and training during extinction. Correlation between the level of extinction and the number of BrdU+ cells in dentate gyrus was analyzed with Pearson correlation test. All data are presented as mean ± SEM.

## Competing interests

The authors declare that they have no competing interests.

## Authors' contributions

H-GK, D-JJ, and JS carried out all the experiments and outlined the manuscripts, designed the studies and wrote the manuscript. J-HC and CK participated in immunohistochemistry and behavioral experiments. Y-SL, HS and Y-HJ participated in designing the study and gamma-ray irradiation. B-KK supervised the experiments, participated in the interpretation of data and wrote the manuscript. All authors read and approved the final manuscript.
